# Contrasting Bacterial and Fungal Assemblages Across Three Co-Flowering Exotic Plant Species Visited by Stingless Bees in Bali, Indonesia

**DOI:** 10.3390/microorganisms14102302

**Published:** 2026-10-09

**Authors:** Joko Pilianto, Yao Xiao, Mochammad Syamsul Hadi, Restu Rizkyta Kusuma, Tita Widjayanti, Amr Abou El-Ela, Syed Shane Alam, Surnaa TabinKhalkha, Kaleem Ullah Shah, Hamdy H. Aly, Xiao Yu, Kamila Muyasarah, Asim Munawar, Wenwu Zhou, Zengrong Zhu, Dun Wang

**Affiliations:** 1 College of Plant Protection, Northwest A&F University, Yangling 712100, China; joko.pilianto@gmail.com (J.P.); yaoxiaofreedom@163.com (Y.X.); shanealam18@gmail.com (S.S.A.); msurna@foxmail.com (S.T.); kaleemullahshahjee@gmail.com (K.U.S.); hamdyaly@nwafu.edu.cn (H.H.A.); yuxiaomichael@foxmail.com (X.Y.); 2 Department of Plant Pests and Diseases, Faculty of Bio-industry, Agriculture and Forestry, Universitas Brawijaya, Jl. Veteran, Malang 65145, Indonesia; msh@ub.ac.id (M.S.H.); restukusuma@ub.ac.id (R.R.K.); 3 The United Graduate School of Agricultural Science, Gifu University, 1-1 Yanagido, Gifu 501-1193, Japan; tita.widjayanti.w2@s.gifu-u.ac.jp; 4 State Key Laboratory of Rice Biology and Breeding, Ministry of Agriculture and Rural Affairs Key Laboratory of Molecular Biology of Crop Pathogens and Insect Pests, Institute of Insect Sciences, Zhejiang University, Hangzhou 310058, Chinawenwuzhou@zju.edu.cn (W.Z.); zrzhu@zju.edu.cn (Z.Z.); 5 Plant Protection Department, Faculty of Agriculture (Saba Basha), Alexandria University, Alexandria 21531, Egypt; 6 Shandong Lushou Seed Industry Co., Ltd., Shouguang 262700, China; 7 Harvard Medical School, Harvard University, Boston, MA 02115, USA; kamila_muyasarah@hms.harvard.edu

**Keywords:** floral microbiome, co-flowering plants, 16S rRNA gene, host specificity, bacterial–fungal comparison, ITS2, host-associated variation, Indonesia

## Abstract

Flowers are transient, chemically selective habitats in which microbial communities assemble rapidly and can influence nectar chemistry and pollinator behavior, yet the bacterial and fungal assemblages of tropical, bee-foraged plants remain largely uncharacterized and are seldom examined in parallel. We characterized whole-flower bacterial and fungal microbiota of three co-flowering plant species, *Antigonon leptopus*, *Coffea arabica* and *Xanthostemon chrysanthus*, growing at a single site in Bali, Indonesia, and visited by the stingless bee *Tetragonula laeviceps*, using paired 16S rRNA and ITS2 amplicon profiling. The three composite samples differed markedly in composition, with fewer than four percent of taxa shared among all three plants despite close spatial proximity and shared climate. Bacterial communities were dominated by *Proteobacteria* and fungal communities were dominated by *Ascomycota* and included lineages characteristic of nectar and of bee-associated environments, notably *Asaia* and other *Acetobacteraceae*, *Pantoea* and *Rosenbergiella*. The two markers did not order the hosts congruently in the samples analyzed: bacterial communities grouped *A. leptopus* with *C. arabica*, whereas fungal communities grouped it with *X. chrysanthus*, a decoupling consistent across clustering, taxon sharing and diversity ranking. These findings indicate that these floral communities differed markedly despite close spatial proximity and shared climate and suggest possible cross-kingdom differences in community organization that warrant examination in replicated designs.

## 1. Introduction

Flowers constitute a distinctive and demanding microbial habitat, available for only a short period, presenting strong osmotic gradients in nectar, containing secondary metabolites and antimicrobial compounds and exposed to ultraviolet radiation and to fluctuating temperature and humidity [1,2,3]. Despite these constraints, floral nectar, petals, anthers and stigmas support characteristic assemblages of bacteria and yeasts that establish rapidly after anthesis and can reach high population densities within the lifespan of a single flower [4,5]. These assemblages are increasingly understood as active participants in plant–pollinator interactions rather than incidental colonists. Microbial metabolism can alter nectar sugar composition, amino acid content, pH, temperature and volatile profile and such changes have been shown to influence the foraging decisions of flower-visiting insects and in turn, patterns of pollen transfer and plant reproductive success [6,7,8].

The composition of floral microbial communities is governed by the interaction between dispersal, which determines which microorganisms arrive at a flower, and environmental filtering, which determines which of those arrivals are able to establish. Microorganisms reach flowers through several routes, including deposition by flower-visiting insects, atmospheric transport, rainfall and contact with surrounding vegetation, and the relative importance of these routes differs among taxa [9,10]. Once arrived, colonists encounter a selective environment defined by the chemical composition of nectar, the physical architecture of the flower, its microclimate and its longevity, all of which vary substantially among plant species [11,12]. Priority effects can further amplify small differences in arrival order, since early colonists modify nectar chemistry in ways that facilitate or exclude subsequent arrivals [13,14]. As a consequence, floral communities are frequently reported to be structured more strongly by host plant identity than by geographic proximity, with co-occurring plant species supporting distinguishable communities despite sharing the same visitor assemblage and environmental conditions [15,16].

Bacteria and fungi differ in several respects that may lead them to respond differently to these assembly processes, most evidently in their principal routes of arrival, since nectar-dwelling bacteria are transferred largely through direct contact with flower-visiting insects whereas many filamentous fungi disperse as airborne spores and are deposited independently of visitation [17,18]. The two groups also differ in osmo-tolerance, growth rate and the time required to establish detectable populations within a short-lived flower [19]. Most surveys of floral microbiota nonetheless target a single marker gene and studies that characterize bacteria and fungi in parallel from the same floral material remain comparatively uncommon [20]. Where both have been examined together, the two kingdoms have not always been found to respond to the same host or environmental properties, which suggests that single-marker surveys may capture only part of the structure present in floral communities [21,22].

Current understanding of floral microbiota derives disproportionately from temperate systems and from a limited number of well-studied plant genera [4,23]. Tropical assemblages, in which many plant species flower simultaneously and are visited by diverse communities of eusocial bees, remain considerably less well characterized. In Indonesia, stingless bees (Apidae: Meliponini) are among the most abundant and ecologically important pollinators, contributing to the pollination of both cultivated and non-cultivated plants and supporting a growing meliponiculture sector [24,25]. Agricultural and peri-urban landscapes across the tropics are increasingly composed of introduced flowering plants, including crops, ornamentals and naturalized species and generalist native pollinators forage extensively on these resources alongside native vegetation. The floral microbiota encountered by such pollinators therefore derives substantially from plant species that are not part of the regional flora, a context that has received little attention. *Tetragonula laeviceps* (Smith, 1857) is widely distributed across the archipelago and forages on a broad range of flowering plants in agricultural and agroforestry landscapes [26,27,28]. For colonies of this and other stingless bee species, flowers constitute a point of repeated contact with the surrounding environment, visited by many individuals over the course of a single flowering period, and are therefore a plausible route by which environmental microorganisms may be encountered and acquired [29,30]. Characterizing the microbial communities present on the flowers such colonies visit is a necessary first step toward evaluating this possibility, yet floral microbiota has rarely been described for tropical plant species in the landscapes where stingless bees forage.

The present study characterized the bacterial and fungal microbiota associated with three co-flowering plant species growing at a single site in Bali, Indonesia, and visited by foraging *T. laeviceps* during the sampling period. *Antigonon leptopus* (Polygonaceae), *Coffea arabica* (Rubiaceae) and *Xanthostemon chrysanthus* (Myrtaceae) differ markedly in floral architecture and in the manner in which floral resources are presented to visitors, yet occur within the same landscape and flower simultaneously, so that variation in microbial community composition could be examined among host plants rather than among locations, elevations or seasons. We expected that: (I) floral microbial communities would differ among co-flowering host species, with only a small proportion of taxa shared among all three, consistent with host identity acting as a principal filter on community composition even where plants occur in close proximity and share the same visitor assemblage; (II) bacterial and fungal communities recovered from the same floral material would not necessarily order these hosts in a congruent manner, given the differences in dispersal route, osmo-tolerance and colonization dynamics that distinguish the two kingdoms. By characterizing both kingdoms in parallel within a tropical, bee-foraged plant assemblage, this study contributes to a broader understanding of how floral microbial communities are structured and of the environmental microbial context in which stingless bees forage.

## 2. Materials and Methods

### 2.1. Study Site and Plant Species

This study characterized the floral bacterial and fungal microbiota of three co-flowering plant species in Karangasem Regency, Bali Island, Indonesia (8°21′ S, 115°26′ E; approximately 1300 m above sea level), and sampling was conducted on 16 February 2026, during the wet season.

**Figure 1:**
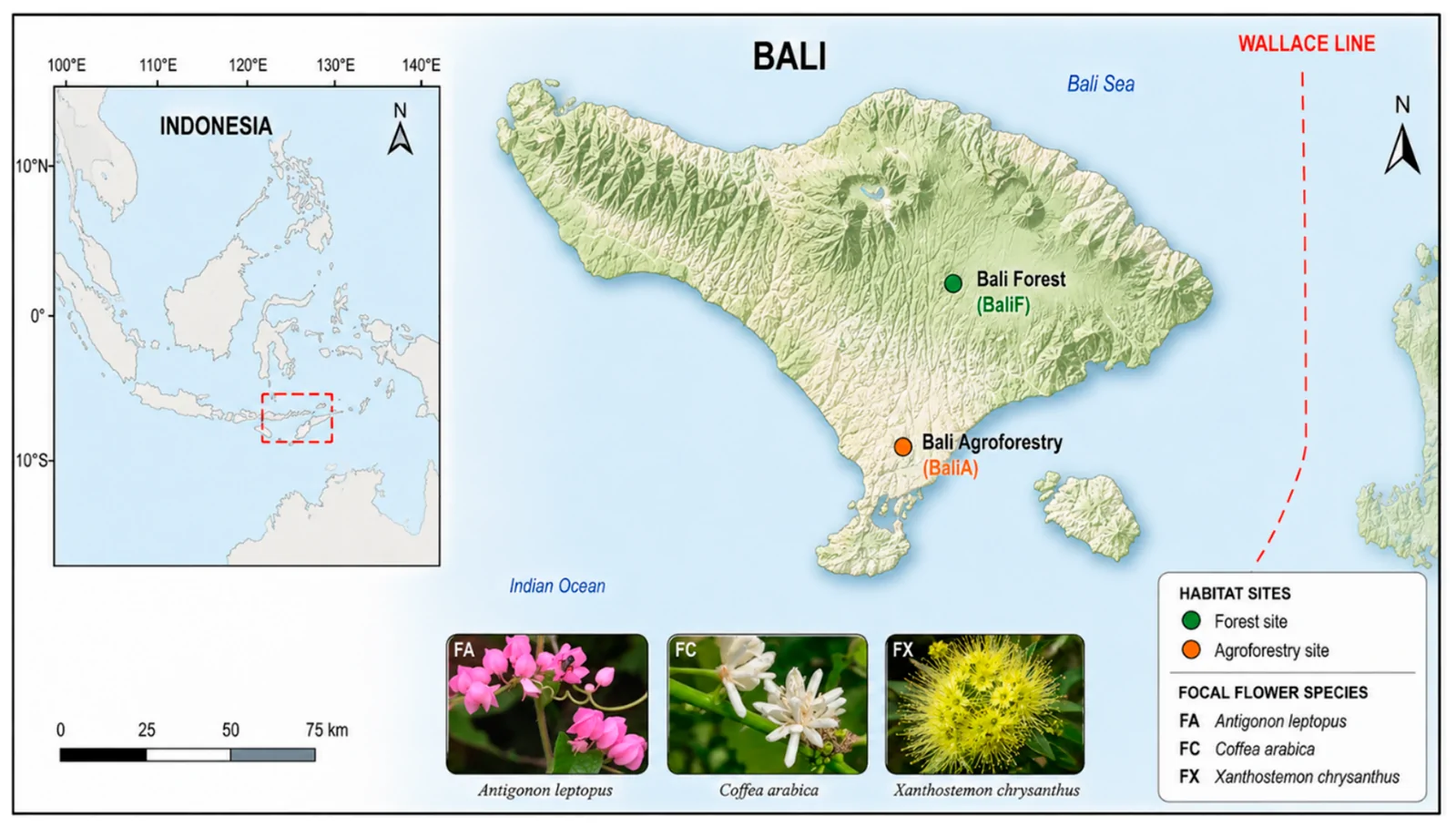
Location of the sampling site in Karangasem Regency, Bali Island, Indonesia, and flowering species *A. leptopus*, *C. arabica*, *X. chrysanthus* phenotypes. Photographs were taken at the sampling site. The red square indicates the location of the study area within Bali.

Three species flowering simultaneously at the site were selected: *Antigonon leptopus* Hook. & Arn. (Polygonaceae), *Coffea arabica* L. (Rubiaceae) and *Xanthostemon chrysanthus* (F. Muell.) Benth. (Myrtaceae), hereafter FA, FC and FX. All three are introduced to Bali. *A. leptopus* is native to Mexico and Central America and occurs at the site as a naturalized, freely reproducing vine; *C. arabica* is native to the Ethiopian highlands and is grown as a smallholder crop under low-input management, with no fungicide application reported by growers for the period preceding sampling; and *X. chrysanthus* is native to northeastern Queensland, Australia, and is planted locally as an ornamental. All three occurred within the foraging range of *T. laeviceps* colonies [31,32] and were observed to be visited by foraging stingless bees during the sampling period. The species differ in floral architecture: *A. leptopus* bears small open flowers with accessible nectar, *C. arabica* bears tubular short-lived flowers and *X. chrysanthus* bears brush-type inflorescences with numerous exposed stamens [33,34] (Figure 1).

The three species also differ in growth form and reproductive biology. *A. leptopus* is a perennial climbing vine that flowers prolifically over extended periods, is self-compatible, sets seed freely outside its native range and is visited by a broad assemblage of generalist insects. *C. arabica* is an allotetraploid, and a predominantly self-pollinating shrub whose flowers open synchronously in short mass-flowering episodes lasting only a few days. *X. chrysanthus* is a canopy tree bearing brush-type inflorescences adapted to generalist insect and bird visitation within its native Queensland range, and is propagated vegetatively for ornamental planting.

### 2.2. Floral Sampling

For each host species, three to four flowers at anthesis were collected from each of five to six individual plants between 08:00 and 12:00 local time (WITA; UTC+8). Sampling was conducted under dry conditions, with no rainfall during collection or in the 24 h preceding it. Flowers were left open to floral visitors prior to collection to allow characterization of the naturally assembled floral microbiota under field conditions. Flowers were excised aseptically using sterile forceps and transferred immediately into sterile microcentrifuge tubes. No surface sterilization was performed in order to retain the naturally associated whole-flower microbiota, including microorganisms occurring on floral surfaces, within floral tissues and associated with pollen, nectar and deposited environmental material. Samples were transported on ice immediately after collection and stored at −80 °C until DNA extraction. For each host species, all flowers collected were pooled to generate a single composite floral sample of 15 to 24 flowers, thereby integrating floral material from different individuals of the same host species. DNA extracted from each composite sample was subsequently used for parallel bacterial 16S rRNA gene and fungal ITS2 amplicon library preparation. The final dataset therefore comprised three composite floral samples, one per host species, from which six amplicon libraries were generated.

### 2.3. DNA Extraction, Amplification and Sequencing

Total genomic DNA was extracted using the DNeasy Plant Mini Kit (QIAGEN, Hilden, Germany) according to the manufacturer’s protocol. Pooled floral material was aseptically homogenized prior to DNA extraction. DNA concentration and purity were assessed spectrophotometrically and DNA integrity was evaluated by 1% agarose-gel electrophoresis prior to amplification. The V3–V4 hypervariable region of the bacterial 16S rRNA gene was amplified using primers 338F (5′-ACTCCTACGGGAGGCAGCA-3′) and 806R (5′-GGACTACHVGGGTWTCTAAT-3′) [35,36]. The fungal internal transcribed spacer 2 (ITS2) region was amplified using primers ITS3 (5′-GCATCGATGAAGAACGCAGC-3′) and ITS4 (5′-TCCTCCGCTTATTGATATGC-3′) [37]. Separate PCR amplifications were performed for the bacterial and fungal markers. Each 25 µL PCR contained 12.5 µL of 2× high-fidelity PCR master mix, 0.5 µL of each primer (10 µM), 2.5 µL of template DNA and 9.0 µL of nuclease-free water. Thermal cycling consisted of an initial denaturation at 95 °C for 5 min, followed by 25 cycles of 95 °C for 30 s, 50 °C for 30 s and 72 °C for 40 s and a final extension at 72 °C for 7 min.

PCR products were verified by agarose-gel electrophoresis and purified using magnetic bead-based purification. Purified amplicons were quantified, indexed by limited-cycle PCR and pooled at equimolar concentrations for sequencing. The bacterial 16S rRNA and fungal ITS2 libraries were sequenced separately on an Illumina NovaSeq 6000 platform (Illumina, San Diego, CA, USA) using 2 × 250 bp paired-end sequencing. Library preparation and sequencing were performed commercially by Biomarker Technologies (BMK) GmbH, Munster, Germany.

### 2.4. Sequence Processing and Taxonomic Annotation

Raw paired-end reads from the bacterial 16S rRNA gene and fungal ITS2 libraries were processed using the same overall bioinformatics workflow, with marker-specific primer sequences and taxonomic reference databases. Initial sequence quality control was performed using Trimmomatic v0.33 [38] and residual primer and adapter sequences were removed using Cutadapt v1.9.1 [39]. Quality-filtered paired-end reads were subsequently processed using the DADA2 algorithm [40] implemented in QIIME 2 (v2020.6) [41]. DADA2 was used for quality filtering, sequence dereplication, error correction, paired-end read merging and chimera removal, resulting in the generation of amplicon sequence variants (ASVs). Quality filtering and read truncation were performed using the default DADA2 parameters implemented in the provider’s standard pipeline, with truncation positions selected automatically from the per-run quality profiles of each marker gene. ASVs represented by fewer than three reads across the dataset were removed prior to downstream analyses. No additional abundance or prevalence filtering was applied and in the absence of negative controls, taxa occurring below 0.1% relative abundance within a library were not used as a basis for ecological interpretation.

Taxonomic classification of representative ASV sequences was performed separately for the bacterial and fungal datasets. Bacterial 16S rRNA gene sequences were assigned against the SILVA release 138 reference database [42], whereas fungal ITS2 sequences were classified against the UNITE database v8.2 [43]. Taxonomic assignments were generated using a combined approach in which representative sequences were classified with a naïve Bayes classifier trained on the respective reference database and cross-checked by BLAST (v2020.6) alignment, with default parameters applied throughout. ASVs classified as chloroplasts, mitochondria, *Archaea* or other non-target lineages were excluded from the bacterial dataset, whereas ASVs not assigned to the kingdom fungi were excluded from the fungal dataset. ASVs confidently assigned to bacteria or fungi but unresolved at lower taxonomic ranks were retained and designated as unclassified at the corresponding rank. Hierarchical taxonomic composition of the resulting communities was additionally visualized using Krona v2.6 [44]. Extraction blanks and PCR no-template controls were not included in the sequencing run; therefore, sequence-based identification of potential reagent-derived contaminants using negative-control prevalence could not be performed.

### 2.5. Diversity Analyses

Bacterial and fungal diversity analyses were performed independently. Alpha diversity was characterized using the number of observed ASVs, abundance-based coverage estimator (ACE), Chao1 richness estimator, Shannon diversity index, Simpson diversity index and Good’s coverage [45]. Simpson diversity was expressed as (1 − D), where ($D=\sum p_{i}^{2}$) and (*p_i_*) represent the proportional abundance of the (i)th ASV. Good’s coverage was used to assess the completeness of sequence sampling. Faith’s phylogenetic diversity (Faith’s PD) was calculated from a rooted phylogenetic tree [46]. Values are presented for both markers, although ITS-based estimates are interpreted with caution given the sequence-length and evolutionary-rate heterogeneity of this region across divergent fungal lineages.

Alpha-diversity metrics were calculated in QIIME 2 v2020.6. Rarefaction curves, including observed-ASV and Shannon diversity rarefaction curves, were generated to evaluate whether sequencing depth adequately captured community diversity. Rank-abundance curves were additionally generated to describe patterns of taxon richness and evenness within each floral community. Beta diversity was evaluated separately for the bacterial and fungal datasets using binary Jaccard dissimilarity, which quantifies compositional differences based on ASV presence or absence [47]. Pairwise Jaccard distance matrices were visualized by principal coordinates analysis (PCoA) and hierarchical clustering using the unweighted pair-group method with arithmetic mean (UPGMA). Pairwise dissimilarities among samples were additionally displayed as clustered heatmaps. Abundance-based ordination was performed by converting ASV counts to relative abundances and applying a Hellinger transformation prior to principal component analysis (PCA), which reduces the influence of highly abundant features and of zero-inflated data [48]. Shared and host-associated ASVs among *A. leptopus*, *C. arabica* and *X. chrysanthus* were quantified separately for the bacterial and fungal datasets and visualized using Venn diagrams generated with the VennDiagram v1.6.20 package in R v4.0.2 [49]. The proportion of ASVs shared among all three host species and those unique to individual hosts was calculated relative to the total number of ASVs detected within the corresponding bacterial or fungal dataset. Because floral material from multiple individuals of each host species was pooled into a single composite sample, diversity indices, ordination patterns, pairwise dissimilarities, clustering and ASV-sharing patterns were interpreted descriptively and no inferential tests of host-species effects were performed.

### 2.6. Functional and Trophic Prediction

The functional potential of bacterial communities was inferred from 16S rRNA ASV sequences using PICRUSt2 v2.3.0-b [50]. Predicted functional profiles were summarized as KEGG orthologs (KOs) and associated pathway categories [51] together with Clusters of Orthologous Groups (COG) functional categories [52]. Ecologically relevant bacterial functions were additionally assigned using FAPROTAX v1.2.4 [53], whereas organism-level phenotypic traits were predicted using BugBase, from which the predicted aerobic phenotype and its contributing bacterial families were examined [54]. Functional and phenotypic profiles were compared descriptively among the three host-associated bacterial communities. Fungal trophic modes and ecological guilds were assigned using FUNGuild v1.1 based on taxonomic annotations [55]. Only assignments with confidence rankings of “Probable” or “Highly Probable” were retained and classified into major trophic modes, including pathotrophs, symbiotrophs and saprotrophs. All bacterial functional and fungal trophic assignments represent predicted functional or ecological potential rather than directly measured activity.

### 2.7. Cross-Kingdom Community Comparison

To assess whether the two marker genes recovered congruent patterns of similarity among host species, bacterial and fungal community organization were compared using host-associated diversity rankings, pairwise community dissimilarities, hierarchical clustering and patterns of shared and unique ASVs. Concordance between the two datasets was evaluated by comparing the relative positioning and clustering of the three host species across the independently generated 16S rRNA and ITS2 community profiles. Because each host species was represented by a single composite floral sample, these cross-kingdom comparisons were interpreted descriptively and no correlation-based analysis linking bacterial and fungal abundances was performed.

### 2.8. Co-Abundance Summary

Co-abundance among the dominant bacterial and fungal genera was summarized using Spearman rank correlation of relative-abundance profiles and displayed as a correlation network constructed from the 50 most abundant genera. Because each marker gene is represented by three composite samples, these displays are presented as a visual summary of the data rather than as an inferred interaction network and no network topology metrics or node-role classifications were derived from them.

### 2.9. Data Visualization

Data visualization was performed using QIIME 2 v2020.6, R v4.0.2 with ggplot2 and VennDiagram and Python v3.8.3 with Matplotlib v3.2.2. Bacterial 16S rRNA and fungal ITS2 datasets were visualized independently, with corresponding analyses presented in parallel to facilitate comparison of community composition, diversity and host-associated patterns between the two microbial kingdoms.

## 3. Results

### 3.1. Sequencing Output and ASV Recovery

Amplicon sequencing generated 200,275 raw paired-end reads from the three bacterial libraries and 240,443 raw paired-end reads from the three fungal libraries. After quality filtering, merging and chimera removal, 144,316 non-chimeric bacterial reads and 208,231 non-chimeric fungal reads were retained for downstream analysis (Table S1). Merged bacterial amplicons fell between 400 and 440 bp, consistent with the expected length of the 16S rRNA gene V3–V4 region, whereas fungal amplicons ranged more broadly from approximately 240 to 460 bp, as expected for the variable-length ITS2 spacer (Figure S1A,B).

**Figure 2:**
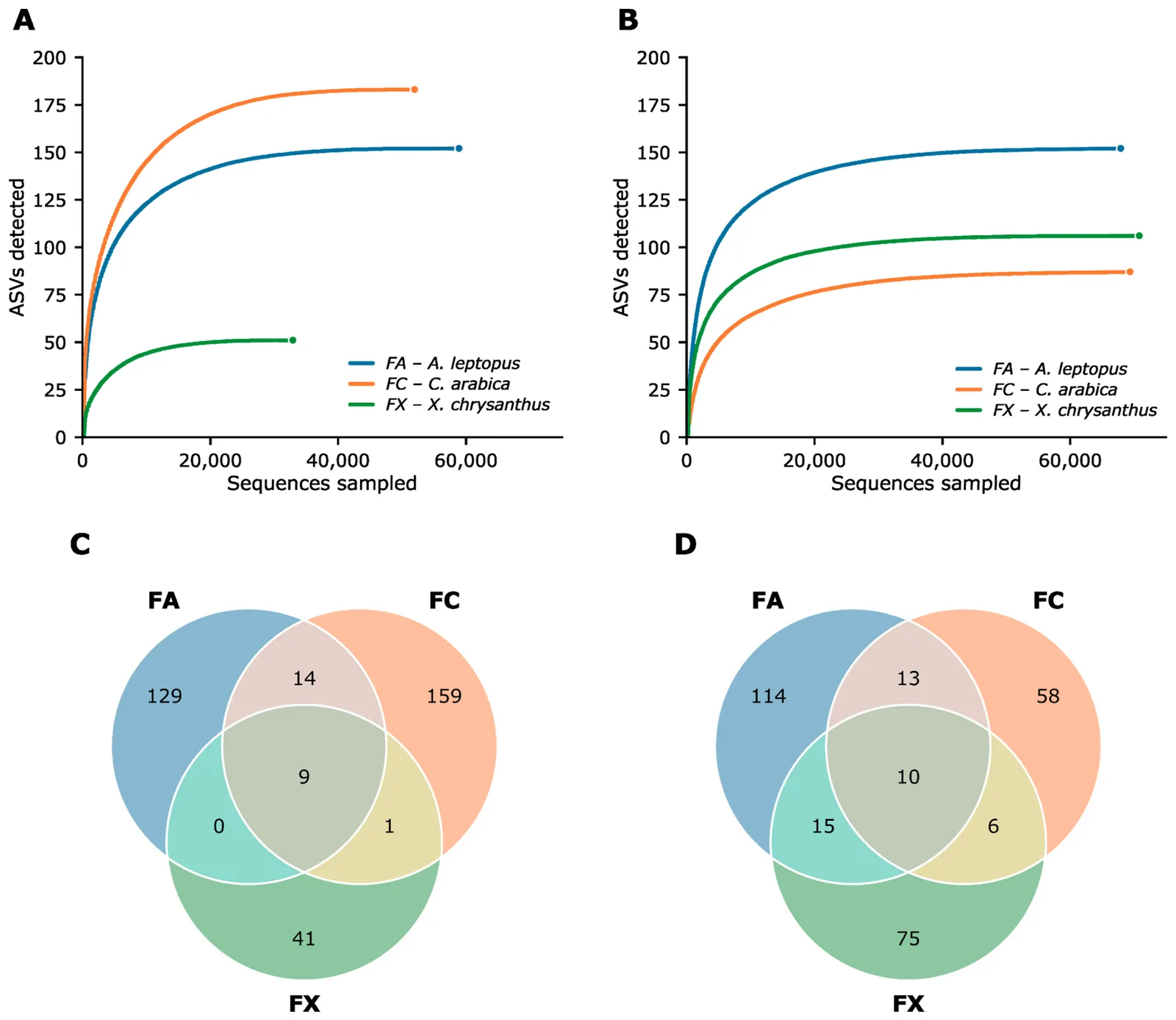
Sequencing sufficiency and ASV sharing among host flowering species. (**A**,**B**) Rarefaction curves for (**A**) bacterial and (**B**) fungal communities, showing amplicon sequence variants recovered against sequencing depth. (**C**,**D**) Venn diagrams of ASV sharing among *A. leptopus* (FA), *C. arabica* (FC) and *X. chrysanthus* (FX) for (**C**) bacteria and (**D**) fungi. Values give the number of ASVs in each partition.

Sequencing depth was uneven among the bacterial libraries, with *A. leptopus* and *C. arabica* yielding 80,030 and 78,844 raw reads, respectively, whereas *X. chrysanthus* yielded 41,401 raw reads. The three fungal libraries were considerably more uniform in this respect, ranging only from 80,107 to 80,181 raw reads. Despite this difference in depth, Good’s coverage reached 1.0000 for all six libraries and rarefaction curves approached an asymptote well within the depth achieved for both marker genes, indicating that sequencing effort was adequate to recover the amplicon sequence variants present in each floral community (Figure 2A,B). Denoising recovered 353 bacterial and 291 fungal amplicon sequence variants in total. Bacterial variant counts were 152 for *A. leptopus*, 183 for *C. arabica* and 51 for *X. chrysanthus*, while the corresponding fungal counts were 152, 87 and 106 (Figure S2). The bacterial library obtained from *X. chrysanthus* therefore combined both the lowest sequencing depth and the lowest variant count, although its coverage value and plateaued rarefaction curve indicate that the reduced depth was nonetheless sufficient to recover the variants present in that flower community.

The great majority of amplicon sequence variants were confined to a single host plant species rather than being shared among the three species examined. Only nine of the 353 bacterial variants (2.5%) and ten of the 291 fungal variants (3.4%) were detected in all three hosts, whereas 329 bacterial variants (93.2%) and 247 fungal variants (84.9%) occurred in one host alone (Figure 2C,D; Table S2). Pairwise sharing between hosts differed markedly between the two marker genes. Among bacteria, *A. leptopus* and *C. arabica* shared 14 variants that were absent from *X. chrysanthus*, whereas no variant at all was shared exclusively between *A. leptopus* and *X. chrysanthus*. Among fungi this pattern was reversed, with *A. leptopus* and *X. chrysanthus* sharing 15 exclusive variants, the largest pairwise overlap recovered for that marker, compared with 13 shared exclusively between *A. leptopus* and *C. arabica*. Because unique variants are disproportionately represented by low-abundance sequences, which are the most sensitive to stochastic detection and to reagent-derived background, these partitions are reported as descriptive counts rather than as evidence of host-restricted taxa.

### 3.2. Taxonomic Composition of Floral Bacterial and Fungal Communities

**Figure 3:**
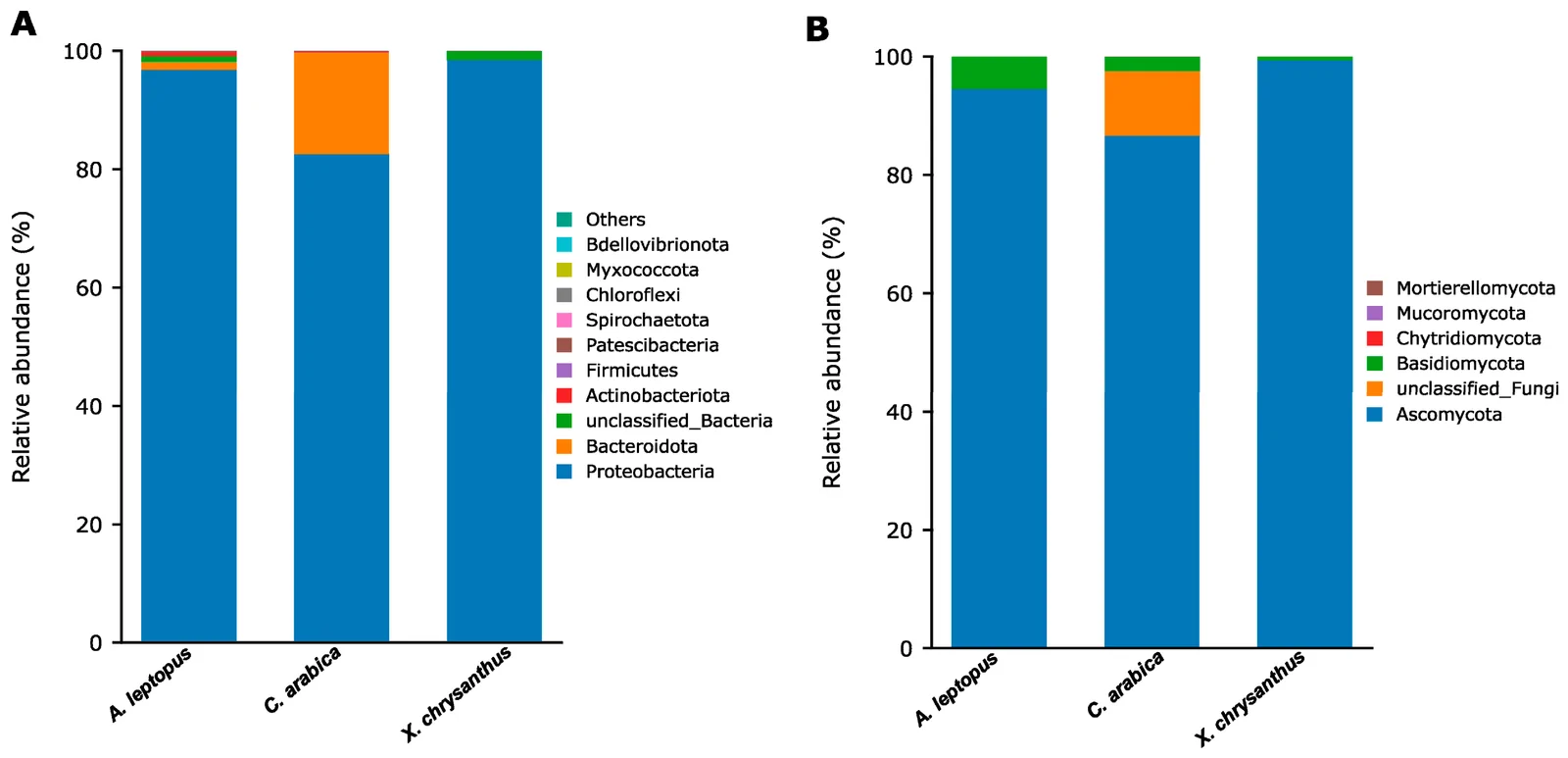
Phylum-level composition of floral bacterial and fungal communities. (**A**) Bacterial and (**B**) fungal phyla across the three host flowering species. Bars show relative abundance; the ten most abundant phyla are shown individually and all remaining phyla are pooled as “Others”. Sequences not assigned below kingdom level are shown as “unclassified_Bacteria” and “unclassified_Fungi”.

Both marker genes recovered communities dominated by a single phylum across all three host plants, with the remaining phyla present at comparatively low relative abundance. *Proteobacteria* accounted for 96.8% of bacterial sequences in *A. leptopus*, 82.5% in *C. arabica* and 98.5% in *X. chrysanthus*, while *Bacteroidota* reached 17.2% in *C. arabica* but remained at 1.3% or below in the other two hosts (Figure 3A). *Actinobacteriota* and unclassified_Bacteria together contributed less than 2% in any host and the remaining phyla, including *Firmicutes*, *Patescibacteria*, *Chloroflexi* and *Spirochaetota*, were each recovered at less than 0.5%. The fungal communities were correspondingly dominated by *Ascomycota*, which represented 94.5%, 86.6% and 99.3% of sequences in *A. leptopus*, *C. arabica* and *X. chrysanthus,* respectively, with *Basidiomycota* contributing between 0.7% and 5.5% and a further 10.9% of fungal sequences in *C. arabica* remaining unclassified below kingdom level (Figure 3B).

**Figure 4:**
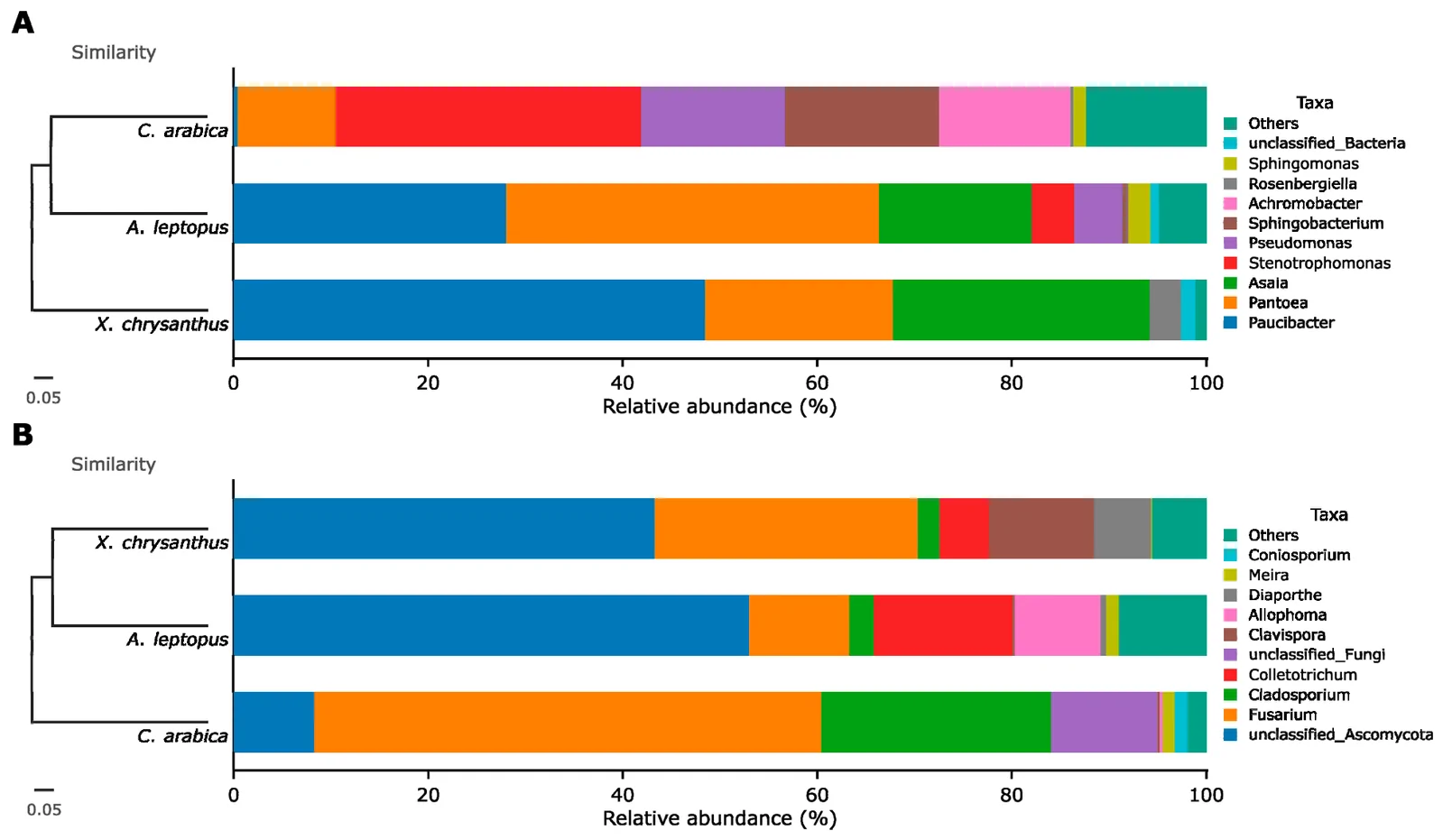
UPGMA clustering and genus-level composition of floral microbial communities. (**A**) Bacterial and (**B**) fungal communities. Dendrogram on the left show hierarchical clustering of the three host flowering species; stacked bars show relative abundance of the ten most abundant genera, with the remainder pooled as “Others”. Scale bars indicate distance.

Compositional differences among hosts were more pronounced at the genus level than at the phylum level. The bacterial communities of *A. leptopus* and *X. chrysanthus* were both dominated by *Paucibacter*, *Pantoea* and *Asaia*, which together accounted for 82.0 and 94.1% of sequences, respectively, whereas these three genera were essentially absent from *C. arabica*. The community recovered from *C. arabica* was instead composed largely of *Stenotrophomonas*, *Sphingobacterium*, *Pseudomonas* and *Achromobacter*, alongside a smaller contribution from *Pantoea* (Figure 4A). Among fungi, unclassified_*Ascomycota* and *Fusarium* were recovered from all three hosts but in contrasting proportions, unclassified_*Ascomycota* reached 53.0% in *A. leptopus* and 43.3% in *X. chrysanthus* but only 8.3% in *C. arabica*, where *Fusarium* instead accounted for 52.1% of sequences. *Cladosporium* was recovered almost exclusively from *C. arabica*, *Colletotrichum* and *Allophoma* were characteristic of *A. leptopus*, and *Clavispora* and *Diaporthe* were largely restricted to *X. chrysanthus* (Figure 4B).

Annotation depth reflected these compositional differences; bacterial sequences were assigned to between 37 and 51 genera per host, with *A. leptopus* yielding the greatest number of genera and *C. arabica* the greatest number of species-level assignments (132). For the fungal communities, *A. leptopus* yielded the highest annotation depth at every rank below class, resolving 76 genera and 95 species, compared with 49 genera in *X. chrysanthus* and 44 in *C. arabica* (Table S3). The same qualitative pattern was evident above ASV level: of the 91 bacterial and 107 fungal genera recovered in total, each host yielded between 37 and 51 bacterial and between 44 and 76 fungal genera, so that a substantial proportion of genera in each community was not recovered from all three hosts (Table S3). Hierarchical clustering of phylum-level relative abundance (Figure S3A,B) and the distribution of representative sequences across the bacterial and fungal phylogenies were consistent with these patterns (Figure S4A,B).

### 3.3. Alpha Diversity and Community Evenness

Alpha-diversity indices varied among the three host plants and for the same set of hosts, followed opposite orders for the two marker genes (Table 1). Bacterial richness and diversity were highest in *C. arabica*, which yielded 183 observed variants and a Shannon index of 4.45, followed by *A. leptopus* (152 variants, Shannon 3.84) and *X. chrysanthus* (51 variants, Shannon 2.37). For the fungal communities this order was reversed, with *X. chrysanthus* returning the highest Shannon index (3.50), followed by *A. leptopus* (3.30) and *C. arabica* (2.53). Simpson diversity followed the same pattern as Shannon for both markers and ACE and Chao1 estimates were identical or near-identical to the observed variant counts, consistent with the Good’s coverage values reported above. Faith’s phylogenetic diversity ranged from 5.62 to 9.05 for bacteria and from 12.47 to 18.72 for fungi and did not fully parallel variant richness in either case (Table 1).

**Figure 5:**
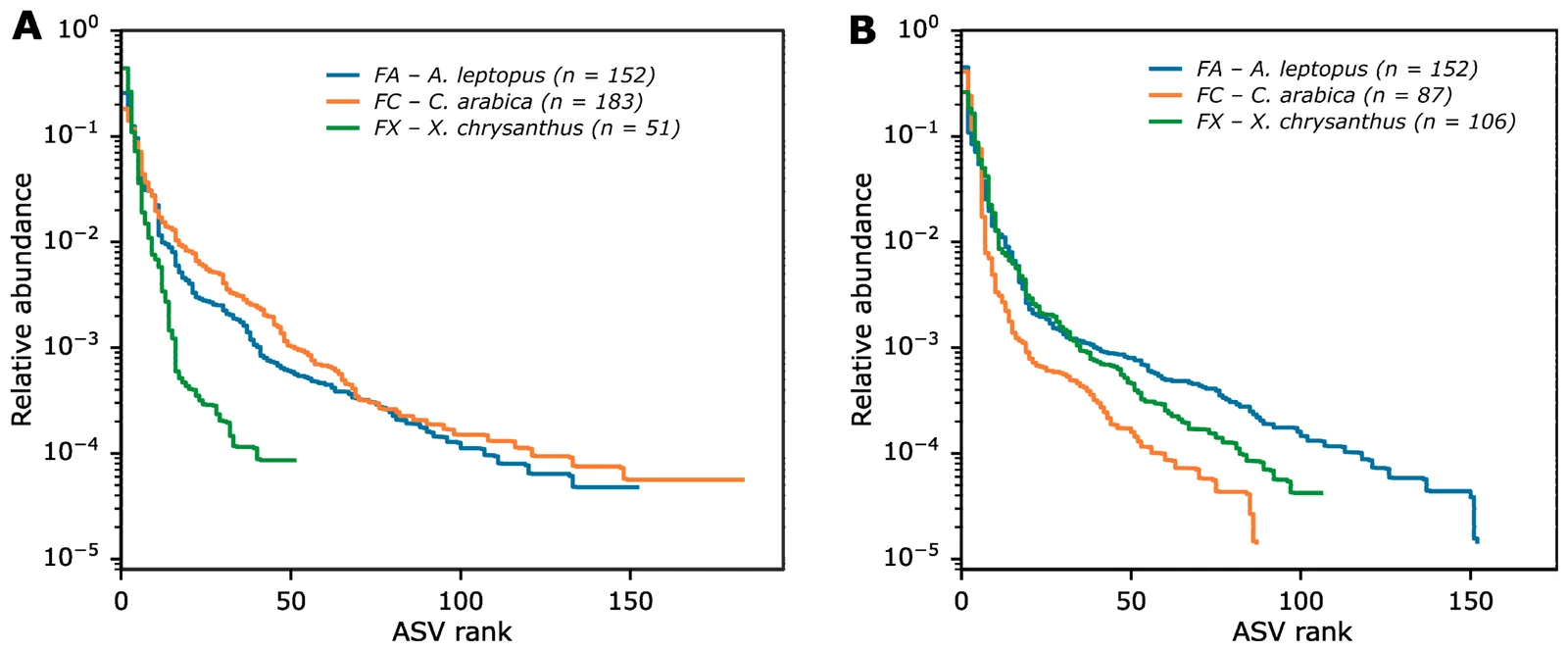
Rank-abundance distributions of floral bacterial and fungal communities. (**A**) Bacterial and (**B**) fungal communities. ASVs are ranked by decreasing relative abundance, plotted on a logarithmic scale. Curve length reflects richness and curve slope reflects evenness.

Rank-abundance curves indicated that these diversity values reflected differences in evenness as well as in richness. The bacterial community of *X. chrysanthus* was both the least rich and the least even of the three, its most abundant variant alone accounting for 43.8% of sequences and its curve declining steeply to a minimum by approximately rank 15. The bacterial curves for *A. leptopus* and *C. arabica* were longer and considerably flatter, their most abundant variants accounting for 25.6% and 18.1% of sequences, respectively (Figure 5A,B). Among fungi, *X. chrysanthus* instead produced the most evenly distributed community of the three, while *C. arabica* showed the steepest decline, its 87 variants spanning a narrower abundance range than those recovered from the other two hosts. Shannon index rarefaction curves stabilized within approximately the first 5000 sequences for all six libraries, indicating that these diversity estimates were not constrained by sequencing depth (Figure S5A,B).

### 3.4. Community Structure Among Host Flowering Species

**Figure 6:**
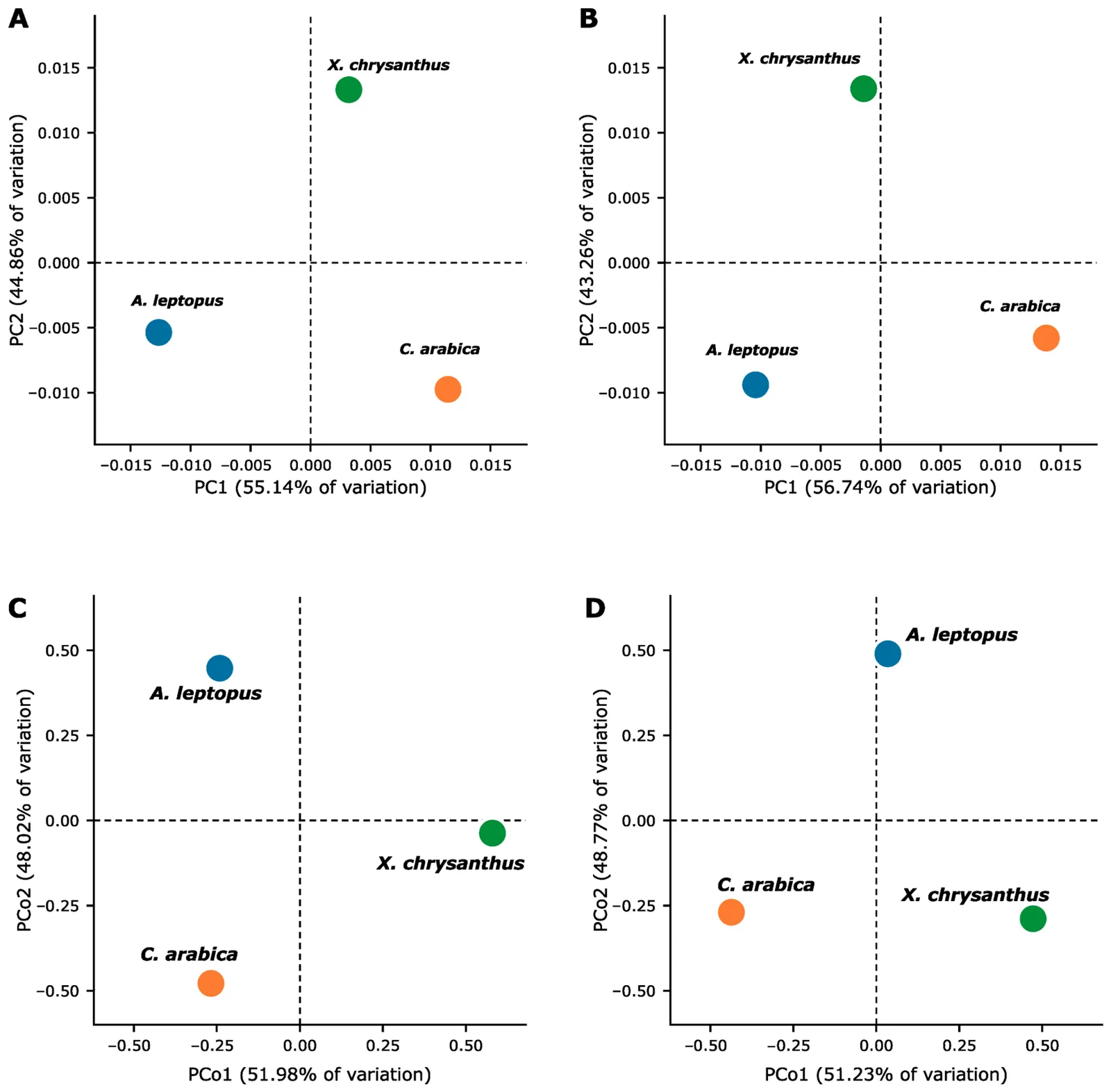
Ordination of floral microbial community composition. (**A**,**B**) Principal component analysis of (**A**) bacterial and (**B**) fungal communities. (**C**,**D**) Principal coordinates analysis of (**C**) bacterial and (**D**) fungal communities. Each point represents one host plant species; axis labels give the percentage of variation attributed to each axis.

Both ordination approaches separated the three floral communities from one another for both marker genes, although they emphasized different aspects of that separation. In the principal component analysis of bacterial abundance profiles, *X. chrysanthus* was positioned apart from the other two hosts along the second component, while *A. leptopus* and *C. arabica* occupied opposite ends of the first component (Figure 6A,B). The principal coordinates analysis of the same communities instead placed *X. chrysanthus* at one extreme of the first coordinate, with *A. leptopus* and *C. arabica* returning similar values on that axis and separating along the second. The fungal ordinations showed a comparable degree of separation among all three hosts. Because each host is represented by a single library, the three points are positioned exactly in two dimensions and the two reported axes necessarily account for all of the variation between them; these ordinations therefore describe the relative positions of the communities analyzed rather than quantifying their separation (Figure 6C,D).

Hierarchical clustering recovered different topologies for the two marker genes. The bacterial communities grouped *A. leptopus* with *C. arabica*, with *X. chrysanthus* joining basally, whereas the fungal communities grouped *A. leptopus* with *X. chrysanthus*, with *C. arabica* joining basally (Figure 4 and Figure S6A,B). These topologies were consistent with the pairwise variant overlaps reported above, in which *A. leptopus* shared 14 exclusive bacterial variants with *C. arabica* and none with *X. chrysanthus*, but shared its largest fungal overlap of 15 exclusive variants with *X. chrysanthus*. In both trees, however, the two merge heights were similar, at 0.484 and 0.543 for bacteria and 0.456 and 0.517 for fungi, so that the basal host joined only approximately 12% deeper than the initial pair. All three communities were therefore close to equidistant under both markers, and the recovered groupings rest on comparatively small differences in shared variant composition. Pairwise distance heatmaps showed the same relationships (Figure S7A,B). The clustering topologies and the genus-level composition described in Section 3.2 emphasize different properties of the same communities. In terms of dominant genera, the bacterial communities of *A. leptopus* and *X. chrysanthus* were the more similar pair, both being composed largely of *Paucibacter*, *Pantoea* and *Asaia*. The clustering, by contrast, is based on the presence and absence of all recovered variants, in which the 183 variants of *C. arabica* overlapped those of *A. leptopus* more extensively than did the 51 variants of *X. chrysanthus*. The two descriptions are therefore complementary, reflecting abundance-weighted similarity among dominant taxa on the one hand and shared occurrence across the full community on the other.

### 3.5. Predicted Functional and Trophic Profiles

**Figure 7:**
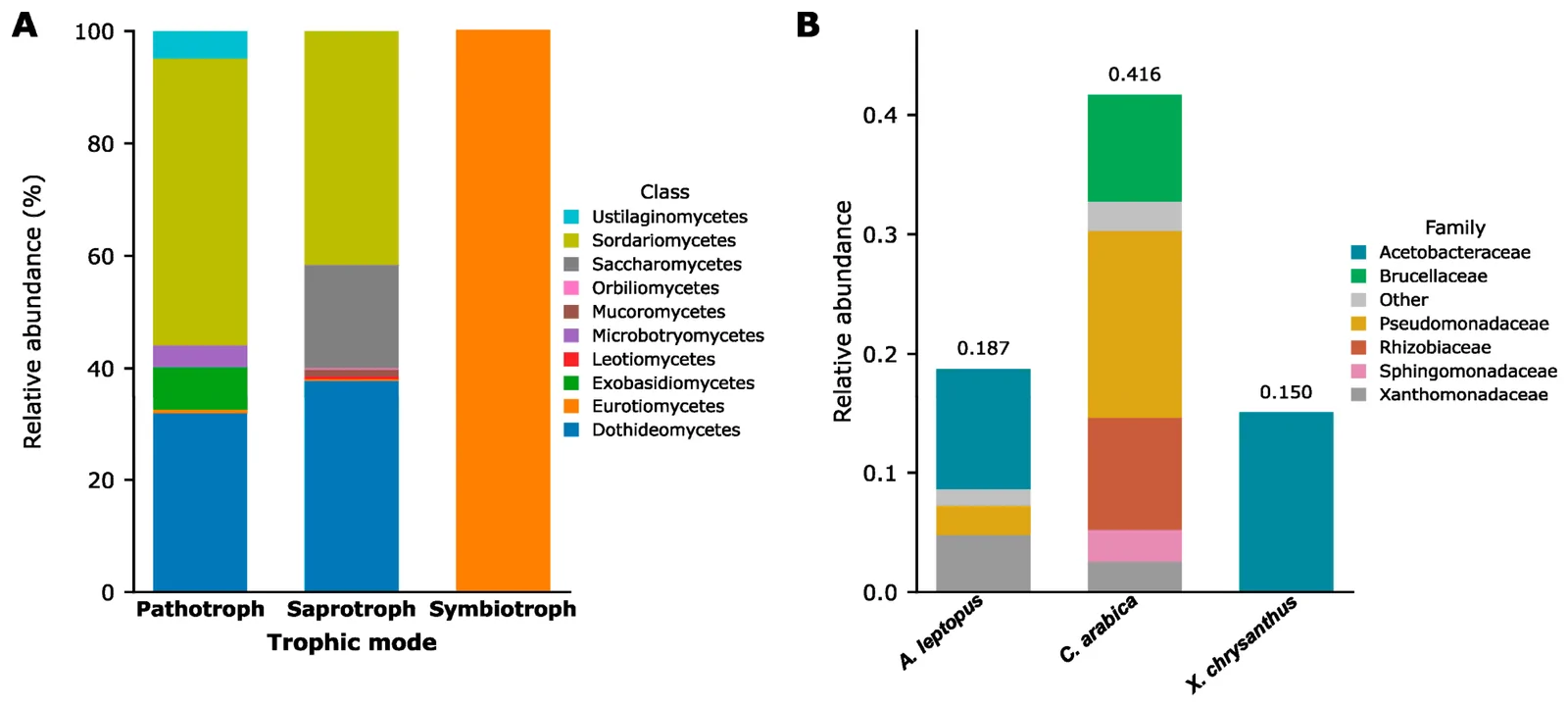
Predicted functional and trophic profiles of floral microbial communities. (**A**) FUNGuild trophic-mode assignment of fungal ASVs, showing class-level composition of the pathotroph, saprotroph and symbiotroph modes. (**B**) BugBase predicted aerobic phenotype of bacterial communities, showing the relative abundance of the trait and the families contributing to it in each host plant species. Values above each bar give the total predicted relative abundance.

Trophic assignment of the fungal communities recovered all three principal modes, with contrasting class-level composition (Figure 7A). Sequences assigned to the pathotroph mode were composed predominantly of *Sordariomycetes* (50.9%) and *Dothideomycetes* (31.7%), with smaller contributions from *Exobasidiomycetes*, *Microbotryomycetes* and *Ustilaginomycetes*. The saprotroph mode showed a broadly similar balance between *Sordariomycetes* (41.7%) and *Dothideomycetes* (37.6%) but included a substantial additional contribution from *Saccharomycetes* (18.4%), whereas the symbiotroph mode was composed entirely of *Eurotiomycetes*. A proportion of fungal sequences could not be assigned to any guild at the confidence levels retained and were therefore excluded from this comparison. The predicted aerobic phenotype of the bacterial communities differed between hosts in both magnitude and family-level composition (Figure 7B). The trait reached a relative abundance of 0.416 in *C. arabica*, compared with 0.187 in *A. leptopus* and 0.150 in *X. chrysanthus*. In *C. arabica* the predicted trait was distributed across several families, principally *Pseudomonadaceae*, *Rhizobiaceae* and *Brucellaceae*, with smaller contributions from *Sphingomonadaceae* and *Xanthomonadaceae*. In *A. leptopus* it was attributable mainly to *Acetobacteraceae* and *Xanthomonadaceae*, while in *X. chrysanthus* it was contributed exclusively by *Acetobacteraceae*, consistent with the prominence of *Asaia* in the genus-level composition of that host.

Gene-family profiles describe the functional categories encoded by a community, inferred here from taxonomic composition by reference to sequenced genomes and were examined to assess whether the compositional differences among hosts corresponded to differences in predicted functional capacity. Predicted gene-family profiles were broadly conserved across the three bacterial communities. Categories associated with metabolism, genetic information processing, environmental information processing and cellular processes were consistently represented in the predicted KEGG pathway profiles (Figure S8A,B) and predicted COG categories relating to metabolism, information storage and processing and cellular processes were likewise distributed across all libraries (Figure S9A,B). Ecological function prediction identified chemoheterotrophy and aerobic chemoheterotrophy as the most abundant inferred categories, together with fermentation, plant-pathogen-associated categories, ureolysis and several nitrogen-cycling functions recovered at lower relative abundance (Figure S10). Variation among the three communities was therefore expressed mainly as differences in the proportional representation of shared predicted categories rather than as the presence of distinct functional repertoires. Co-abundance relationships among the dominant genera of each marker gene are summarized in Figure S11A,B.

## 4. Discussion

### 4.1. Floral Microbiota in Tropical Pollinator Systems

Flowers support distinctive microbial communities that occupy a transient, chemically demanding and repeatedly disturbed habitat, characterized by high osmotic pressure, secondary metabolites, ultraviolet exposure and a short period of availability [3,4]. These communities are increasingly recognized as active participants in plant–pollinator interactions rather than passive passengers, with nectar-inhabiting bacteria and yeasts capable of altering sugar composition, volatile profiles and consequently visitation behavior [6,7,8]. Because flowers are visited by many individual insects during a single flowering period, they also constitute a shared surface across which microorganisms may be transmitted among visitors and between visitors and plants [29,30]. Most of what is known about floral microbial communities derives from temperate systems and from a relatively small number of well-studied plant genera [23]. Tropical assemblages, in which many plant species flower simultaneously and are visited by diverse and largely eusocial bee communities, remain considerably less well characterized. Studies that survey bacteria and fungi in parallel from the same floral material are rarer still, despite evidence that the two kingdoms differ in dispersal mode, growth requirements and tolerance of the nectar environment [17,19]. The present study addressed this gap by characterizing bacterial and fungal communities from three co-flowering plant species growing at a single site in Bali, thereby holding island, region, elevation and season constant while allowing host identity to vary.

### 4.2. Marked Compositional Differences Among the Three Floral Communities

Only a small minority of variants was shared among all three hosts, while the great majority was confined to a single host species. Floral communities growing within meters of one another, under identical climatic conditions and observed visitation by *T. laeviceps*, therefore differed substantially in composition. Co-occurring plant species have frequently been reported to support distinguishable floral microbial communities, with only a small proportion of taxa shared among them [56,57,58]. Several non-mutually exclusive processes may contribute. Nectar differs among plant species in sugar concentration and composition, amino acid content, pH and secondary metabolites and these properties act as a selective filter on which arriving microorganisms establish [59,60]. Floral architecture governs physical accessibility and microclimate and the three species examined here differ markedly in this respect. These differences extend beyond flower shape to the duration and synchrony of flowering, the quantity and accessibility of nectar and the breadth of the visitor assemblage each species attracts, all of which influence how often flowers are contacted and how long arriving microorganisms have to establish. Flower longevity determines the time available for colonization and competitive sorting [61,62] and management history may also contribute, since *C. arabica* was under active cultivation whereas the other two species were not and differences in pruning, shading, fertilization and soil disturbance can alter the microbial environment surrounding flowers. All three species are additionally introduced to Bali rather than components of the native flora, so the assemblage examined here reflects a human-modified landscape in which a native generalist pollinator forages largely on exotic floral resources. Because these species occur outside their native ranges, the floral microbiota recovered here need not resemble those of conspecific populations in their regions of origin, where different environmental microbial pools and pollinator assemblages operate.

### 4.3. Recovered Taxa Are Characteristic of Floral Habitats

The lineages recovered here correspond closely to those reported from floral habitats elsewhere. *Proteobacteria* dominated all three bacterial communities, a pattern consistently reported from nectar and floral surfaces and generally attributed to osmo-tolerance and to the capacity to metabolize the simple sugars predominating in nectar [1,4]. *Pantoea*, recovered from all three hosts, and *Rosenbergiella* are among the most frequently reported nectar-associated genera [2], while *Asaia* and other *Acetobacteraceae* are characteristic of sugar-rich niches and widely reported from nectar, honey and insect-associated environments [19]. The prominence of *Stenotrophomonas*, *Sphingobacterium* and *Pseudomonas* in *C. arabica* is consistent with reports from the phyllosphere of cultivated plants [22,58]. The fungal communities were similarly dominated by *Ascomycota* [16,22]. *Cladosporium* and *Fusarium* are among the most widespread airborne and phyllosphere fungal genera [63], while *Colletotrichum* and *Diaporthe* are commonly recovered as foliar endophytes [64]. *Clavispora*, recovered largely from *X. chrysanthus*, belongs to the *Saccharomycetes*, the class containing the most osmo-tolerant nectar-inhabiting yeasts [65]. The appreciable proportion of sequences unassigned below phylum or kingdom level, particularly among fungi, reflects the incomplete representation of floral and phyllosphere taxa in current reference databases [66].

### 4.4. Bacterial and Fungal Communities Grouped the Same Hosts Differently

Bacterial and fungal communities recovered from the same floral material did not order the three host plants in the same way, a contrast evident in the clustering topology, the pairwise variant overlaps and the diversity rankings alike. That the three descriptions derived from different properties of the data should agree suggests the pattern is unlikely to be an artifact of any single analysis, although it is reported here as an observed pattern within these samples rather than as a general ecological property.

Several processes could generate such a decoupling; bacteria and fungi differ in their principal routes of arrival at flowers, with nectar-dwelling bacteria transferred largely by flower-visiting insects whereas many filamentous fungi disperse as airborne spores and are deposited independently of visitation [4,18]. If bacterial arrival is dominated by shared insect vectors and fungal arrival by atmospheric deposition, the two kingdoms might respond to different features of the floral environment and communities resembling one another in one kingdom need not do so in the other. The two groups also differ in tolerance of the nectar environment, in growth rate and in the time required to establish within the lifespan of a single flower [19,61]. Fungal communities may additionally retain a stronger signal of the surrounding vegetation and of the host phyllosphere, decoupling them from the visitation-mediated processes structuring bacterial communities [21,63]. This interpretation requires qualification; in both clustering analyses the two merge heights were similar, so that the basal host joined only slightly deeper than the initial pair, leaving all three communities close to equidistant under both markers. The recovered topologies therefore rest on modest differences in shared variant composition and while their consistency with pairwise overlap and diversity ranking strengthens confidence in the pattern, it should be regarded as a hypothesis for testing rather than an established difference in community assembly between the two kingdoms.

### 4.5. Floral Microbiota as a Potential Environmental Source Pool for Stingless Bees

All three plant species examined here were visited by foraging stingless bees during the sampling period, and *T. laeviceps* is widely distributed in Indonesian agricultural and agroforestry landscapes, where it forages on a broad assemblage of cultivated and non-cultivated flowering plants [24,26,27]. Flowers are consequently a point of repeated contact between colonies and the wider environment, visited for nectar and pollen by many individuals over the course of a single flowering period [29,30]. As a broad generalist, *T. laeviceps* forages across whatever floral resources are locally available and in landscapes such as this one those resources are predominantly introduced species. Several of the bacterial lineages recovered here are among those most consistently reported from bee-associated niches. *Acetobacteraceae*, including *Asaia*, are characteristic of sugar-rich environments and have been repeatedly detected in nectar, honey, beebread and the gut of stingless bees and other corbiculate bees [19,67,68]. *Asaia* was a major constituent of the bacterial communities of *X. chrysanthus* and *A. leptopus,* and *Acetobacteraceae* contributed the entire predicted aerobic phenotype of the former. *Rosenbergiella* and *Pantoea*, both recovered here, are similarly well documented from floral nectar and from flower-visiting insects [1,2]. The floral communities characterized in this study therefore contain lineages overlapping those described from stingless bee gut and hive environments, consistent with the view that floral resources represent one plausible environmental reservoir from which such taxa may be acquired [29,69]. This interpretation nonetheless requires caution, since bees were not sampled and no directional inference can be drawn from taxonomic overlap alone. Shared lineages may equally reflect deposition of gut or hive-derived microorganisms onto flowers during foraging, acquisition by foragers from previously visited flowers or independent colonization of both compartments from a common environmental pool [69].

### 4.6. Predicted Functional Profiles

Functional predictions are treated here as secondary to the community-composition results. Predicted functional profiles indicated that the three bacterial communities shared a broadly conserved set of inferred gene-family categories, with variation expressed mainly as differences in proportional representation rather than as distinct functional repertoires. Predicted ecological functions were dominated by chemoheterotrophy and aerobic chemoheterotrophy, consistent with the sugar-rich, aerobic character of the floral environment [19]. Fungal trophic assignment recovered pathotroph, saprotroph and symbiotroph modes, with the saprotroph mode including a substantial contribution from *Saccharomycetes*, the class containing the most nectar-inhabiting yeasts [65]. These inferences should be interpreted with restraint; all functional and trophic assignments were derived from taxonomic annotation against reference databases rather than from measured gene content or metabolic activity and their accuracy depends on how well the taxa present are represented in those databases. Predictive accuracy declines appreciably for communities from environments under-represented in reference genome collections [70], and guild annotations for many floral and phyllosphere fungi remain incomplete [66]. The predicted profiles are therefore best regarded as descriptions of the functional potential implied by community composition and as a basis for hypotheses requiring metagenomic, meta-transcriptomic or metabolomic approaches to test.

### 4.7. Limitations and Future Directions

The design adopted here characterized floral microbial communities under natural field conditions while holding site, climate and season constant, and the inferences apply at that scale. With each host species represented by a single composite sample per marker gene, the comparisons reported are descriptive contrasts among the libraries analyzed rather than generalizations to host populations, and floral communities are additionally known to vary with flower age, weather, rainfall and visitation history [62,71]. Host identity is further confounded with plant family and cultivation status. As floral surfaces are a low-biomass substrate in which reagent-derived sequences can form an appreciable proportion of the recovered community [72], taxa below 0.1% relative abundance were not used for ecological interpretation, and the counts of host-restricted variants are correspondingly the least robust element of the comparison. Floral traits and reproductive outcomes were not quantified in this study, so the microbial patterns described here cannot be related directly to pollination success; doing so would require paired measurement of visitation, pollen transfer and seed set alongside microbial sampling. Establishing whether the cross-kingdom decoupling observed here is a general property of tropical floral microbiota will require sampling across broader co-flowering assemblages, while paired characterization of flowers and of the foragers visiting them would allow the contribution of floral resources to bee-associated communities to be resolved directly. Applied to managed and semi-managed landscapes, the paired bacterial and fungal approach used here provides a means of characterizing the floral microbial environment encountered by pollinators, with relevance to both crop production systems and pollinator ecology.

## 5. Conclusions

Floral microbial communities associated with three co-flowering plant species in Bali differed markedly in composition, with fewer than four percent of amplicon sequence variants shared among all three hosts despite their close spatial proximity, shared climate and observed visitation by *T. laeviceps*. Bacterial and fungal communities recovered from the same floral material did not order these hosts in the same way, a decoupling expressed consistently in clustering topology, pairwise variant sharing and diversity ranking and suggesting possible cross-kingdom differences in community organization. Several of the lineages detected, notably *Asaia* and other *Acetobacteraceae*, *Pantoea* and *Rosenbergiella*, are among those commonly reported from nectar and from bee-associated environments, consistent with the view that flowers constitute one plausible environmental reservoir contributing to the microbiota of stingless bees foraging within these landscapes. These findings identify co-flowering plant assemblages as an informative and underexplored compartment of tropical pollinator ecology and suggest that characterizing bacteria and fungi in parallel may capture aspects of floral community structure that single-marker surveys would not resolve.

## Figures and Tables

**Table 1:** Alpha-diversity indices of floral bacterial and fungal communities.

Marker/Host Species	ASVs	ACE	Chao1	Simpson (1 − D)	Shannon	Faith’s PD	Good’s Coverage
16S—*A. leptopus* (FA)	152	152.00	152.0	0.8686	3.8437	9.0469	1.0000
16S—*C. arabica* (FC)	183	183.00	183.0	0.9137	4.4500	8.5083	1.0000
16S—*X. chrysanthus* (FX)	51	51.00	51.0	0.7048	2.3666	5.6173	1.0000
ITS2—*A. leptopus* (FA)	152	152.44	153.0	0.7569	3.3035	18.7233	1.0000
ITS2—*C. arabica* (FC)	87	87.44	87.5	0.7389	2.5308	12.4683	1.0000
ITS2—*X. chrysanthus* (FX)	106	106.00	106.0	0.8531	3.5039	13.6331	1.0000

Note: Each value derives from a single pooled library per host species, so these indices describe the individual samples analyzed and are not accompanied by measures of dispersion or by tests of difference among host species.

## Data Availability

The raw 16S rRNA gene and ITS2 amplicon sequencing reads generated in this study have been deposited in the NCBI Sequence Read Archive under BioProject accession PRJNA1491228 (BioSample accessions SAMN63167978–SAMN63167983). The ASV abundance tables, representative ASV sequences, taxonomic assignment tables and sample metadata are provided as Supplementary Materials. Further inquiries can be directed to the corresponding authors.

## Supplementary Materials

The following supporting information can be downloaded at https://www.mdpi.com/article/10.3390/microorganisms14102302/s1, Table S1. Sequencing depth and ASV recovery for the floral samples; Table S2. Distribution of ASVs among the three host flowering species; Table S3. Number of distinct taxa annotated at each taxonomic rank; Figure S1. Length distribution of effective reads; Figure S2. Number of ASVs recovered per host flowering species; Figure S3. Phylum-level relative abundance with hierarchical clustering; Figure S4. Community distribution mapped onto phylogenetic trees of representative sequences; Figure S5. Shannon index rarefaction curves; Figure S6. UPGMA clustering trees; Figure S7. Sample-distance clustering heatmaps; Figure S8. Predicted KEGG functional profiles of floral bacterial communities; Figure S9. PICRUSt2-predicted COG functional profiles of floral bacterial communities; Figure S10. Predicted ecological functions of floral bacterial communities; Figure S11. Spearman correlation networks among dominant genera.

## Abbreviations

The following abbreviations are used in this manuscript:

ACE	Abundance-Based Coverage Estimator
ASV	Amplicon Sequence Variant
COG	Clusters of Orthologous Groups
DADA2	Divisive Amplicon Denoising Algorithm 2
FAPROTAX	Functional Annotation of Prokaryotic Taxa
FUNGuild	Fungal Functional Guild
ITS2	Internal Transcribed Spacer 2
KEGG	Kyoto Encyclopedia of Genes and Genomes
KO	KEGG Ortholog
PCA	Principal Component Analysis
PCoA	Principal Coordinates Analysis
PCR	Polymerase Chain Reaction
PD	Phylogenetic Diversity
PICRUSt2	Phylogenetic Investigation of Communities by Reconstruction of Unobserved States 2
QIIME2	Quantitative Insights Into Microbial Ecology 2
SILVA	SILVA Ribosomal RNA Gene Database
UPGMA	Unweighted Pair-Group Method with Arithmetic Mean
WITA	Waktu Indonesia Tengah (Central Indonesian Time)
UNITE	UNITE Fungal ITS Database
rRNA	Ribosomal RNA
