# Supplementary material for: Contrasting Bacterial and Fungal Assemblages Across Three Co-Flowering Exotic Plant Species Visited by Stingless Bees in Bali, Indonesia
Source: Microorganisms. 2026 Oct 9;14(10):2302. doi: 10.3390/microorganisms14102302 (PMC13707677; doi:10.3390/microorganisms14102302)
Supplement: Supplementary file 1 [file microorganisms-14-02302-s001.zip › microorganisms-4570222-supplementary.pdf]

Supporting Information’s

Contrasting Bacterial and Fungal Assemblages Across Three Co-Flowering Exotic Plant Species Visited by Stingless Bees in Bali, Indonesia

The following supporting information’s are available;

Table S1. Sequencing depth and ASVs recovery for the floral samples.

Marker / host species (code)	Raw reads	Clean reads	Denoised reads	Merged reads	Non-chimeric reads	ASVs	Feature reads
16S – *A. leptopus* (FA)	80,030	71,689	71,538	70,734	59,102	152	58,889
16S – *C. arabica* (FC)	78,844	67,982	67,847	66,609	52,217	183	51,957
16S – *X. chrysanthus* (FX)	41,401	33,876	33,849	33,709	32,997	51	32,901
**16S – total**	200,275	173,547	173,234	171,052	144,316	353	143,747
ITS2 – *A. leptopus* (FA)	80,181	72,955	72,874	72,395	67,988	152	67,898
ITS2 – *C. arabica* (FC)	80,155	72,884	72,813	72,744	69,391	87	69,347
ITS2 – *X. chrysanthus* (FX)	80,107	73,002	72,981	72,763	70,852	106	70,806
**ITS2 – total**	240,443	218,841	218,668	217,902	208,231	291	208,051

Note: the 16S library for *X. chrysanthus* (FX) was sequenced to approximately half the depth of the other two libraries.

Table S2. Distribution of ASVs among the three host flowering species.

ASV partition	Bacteria (16S)	% of bacterial ASVs	Fungi (ITS2)	% of fungal ASVs
A. leptopus only (FA)	129	36.5	114	39.2
C. arabica only (FC)	159	45.0	58	19.9
X. chrysanthus only (FX)	41	11.6	75	25.8
FA ∩ FC only	14	4.0	13	4.5
FA ∩ FX only	0	0.0	15	5.2
FC ∩ FX only	1	0.3	6	2.1
Shared by all three (core)	9	2.5	10	3.4
Total ASVs recovered	353	100	291	100

Note: no bacterial ASV was detected in *A. leptopus* and *X. chrysanthus* without also being present in *C. arabica*, whereas 15 fungal ASVs were shared exclusively by *A. leptopus* and *X. chrysanthus*.

Table S3. Number of distinct taxa annotated at each taxonomic rank.

Taxonomic rank	16S FA	16S FC	16S FX	16S total	ITS2 FA	ITS2 FC	ITS2 FX	ITS2 total
Kingdom	1	1	1	1	1	1	1	1
Phylum	9	5	7	11	4	5	4	6
Class	10	7	10	15	13	14	14	17
Order	22	17	21	34	26	22	25	33
Family	31	27	28	51	48	33	38	69
Genus	51	46	37	91	76	44	49	107
Species	114	132	47	247	95	53	69	153

Note: the two marker genes rank the host species differently. *C. arabica* yielded the most bacterial species-level annotations (132) but not the most bacterial genera (46 versus 51 for *A. leptopus*), whereas *A. leptopus* yielded the most fungal taxa at every rank below class.

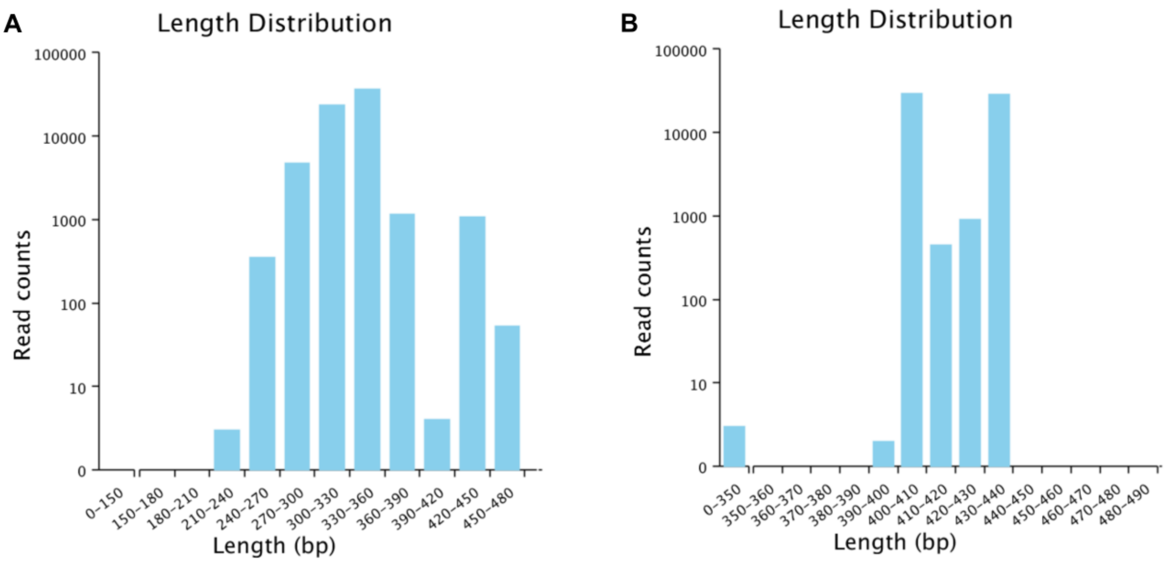

Figure S1. Length distribution of effective reads. (**A**) Bacterial and (**B**) fungal libraries. The x-axis gives sequence length in base pairs and the y-axis the number of reads, plotted on a logarithmic scale.

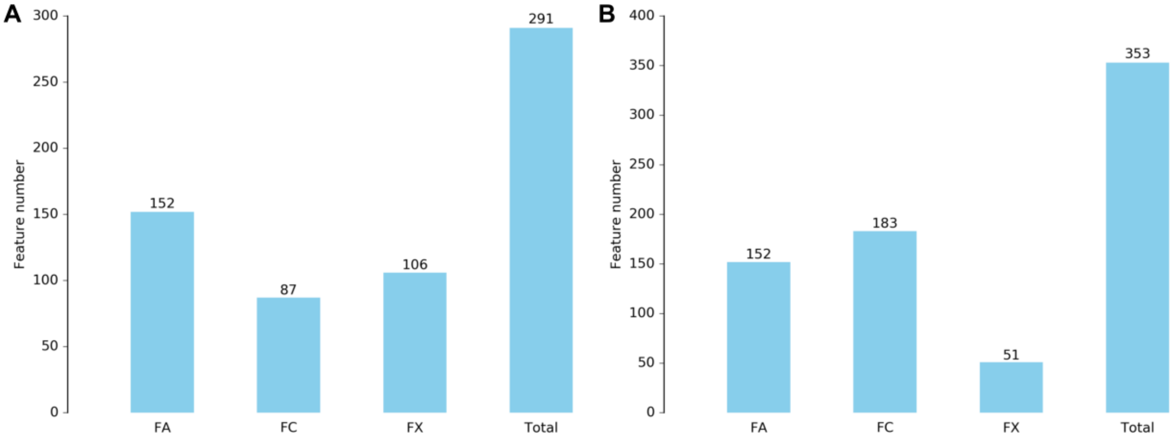

Figure S2. Number of ASVs recovered per host flowering species. (**A**) Bacterial and (**B**) fungal communities. Values above each bar give the number of amplicon sequence variants; "Total" gives the number recovered across all three host flowering species combined.

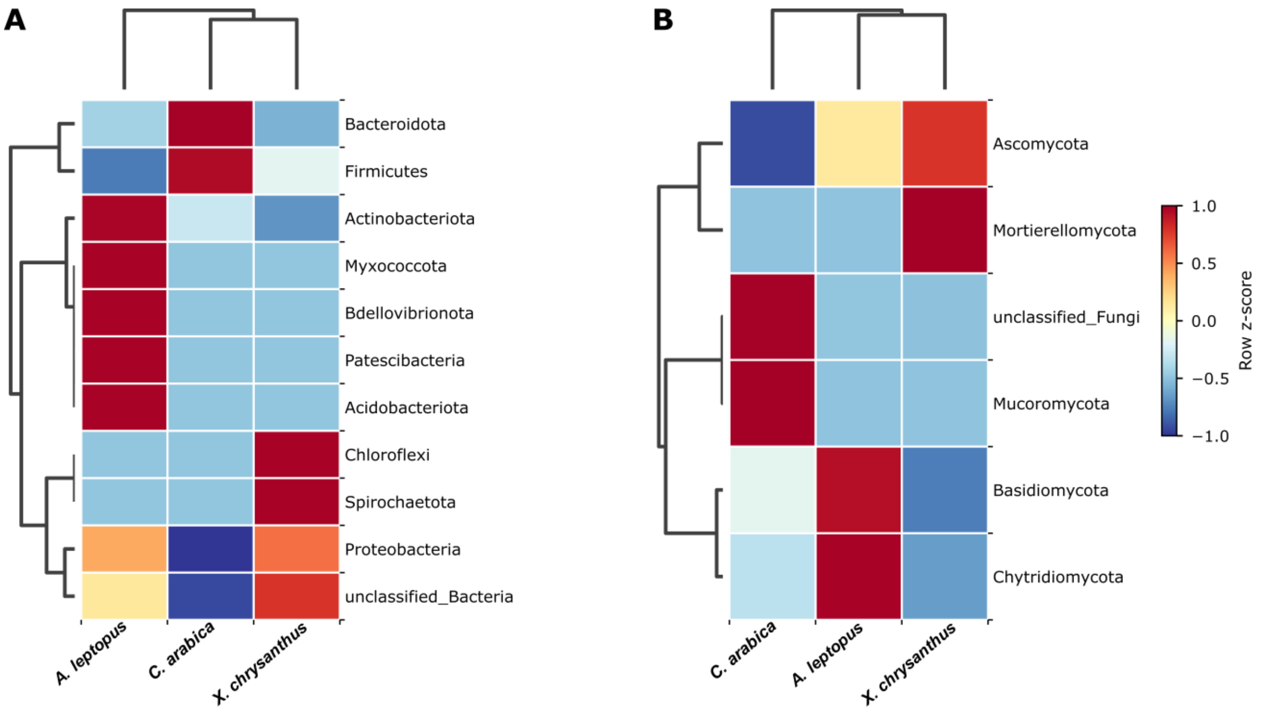

Figure S3. Phylum-level relative abundance with hierarchical clustering. (**A**) Bacterial and (**B**) fungal communities. Color indicates the row z-score of relative abundance, so values are comparable across host plant species within a phylum but not between phyla. Dendrogram cluster phyla by abundance profile (rows) and host plant species by composition (columns).

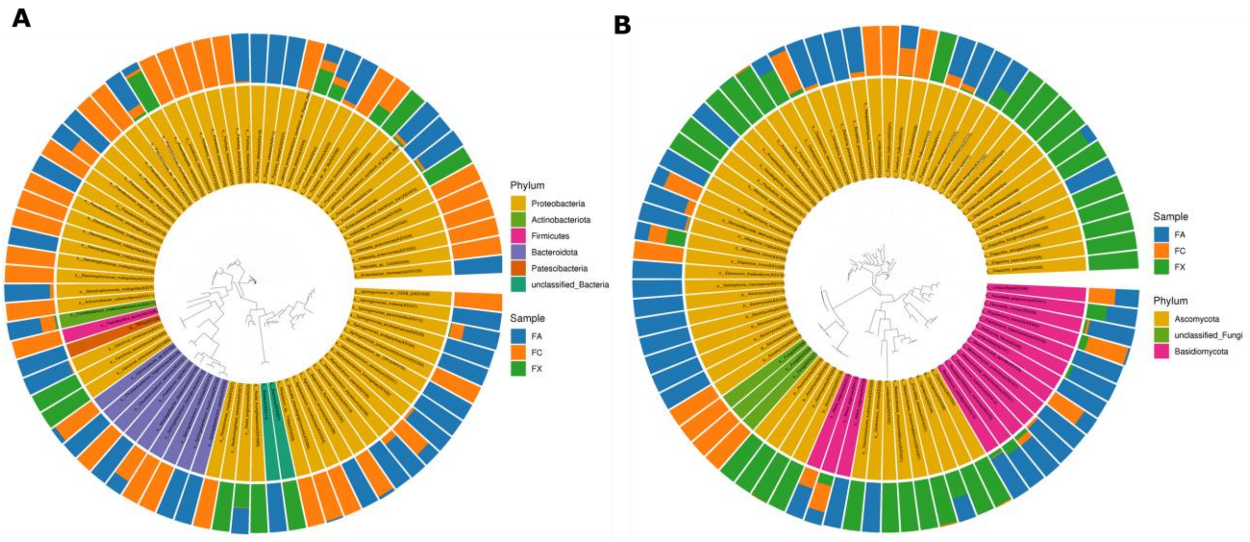

Figure S4. Community distribution mapped onto phylogenetic trees of representative sequences. (**A**) Bacterial and (**B**) fungal communities. The inner ring colors branches by phylum and the outer ring shows the distribution of each taxon across the three host flowering species.

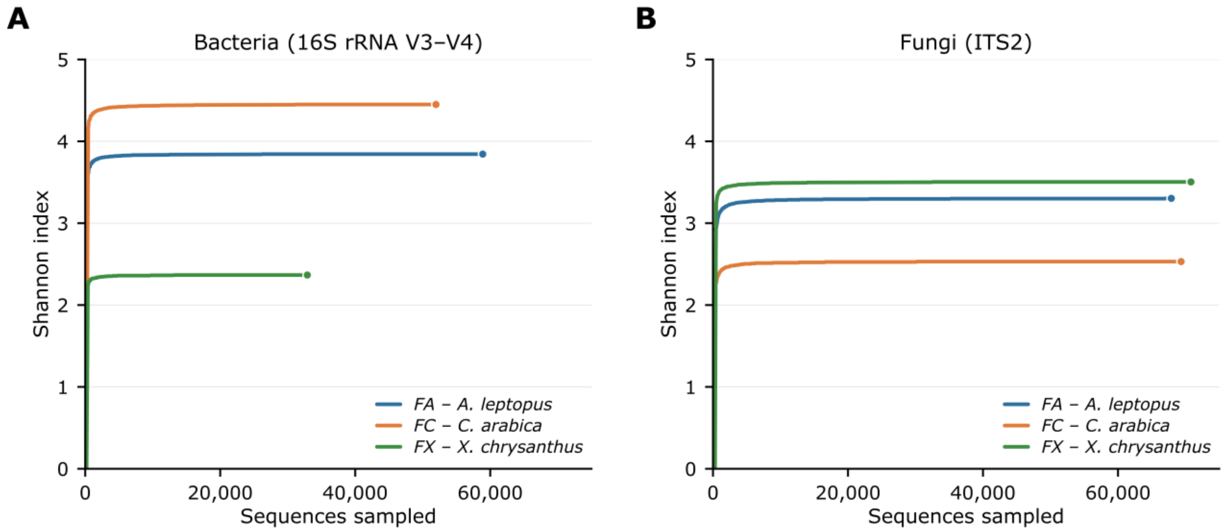

Figure S5. **Shannon index rarefaction curves.** (**A**) Bacterial and (**B**) fungal communities, showing the Shannon index against sequencing depth.

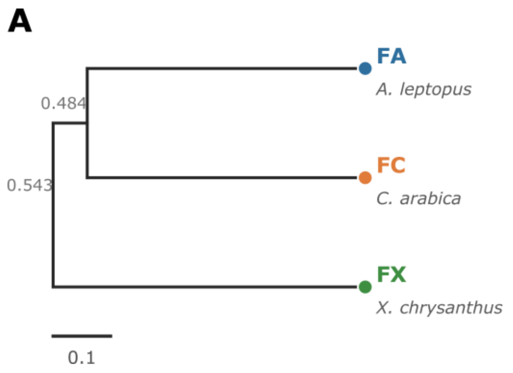

Figure S6. UPGMA clustering trees. (**A**) Bacterial and (**B**) fungal communities. Values at each node give the merge height; scale bars indicate distance.

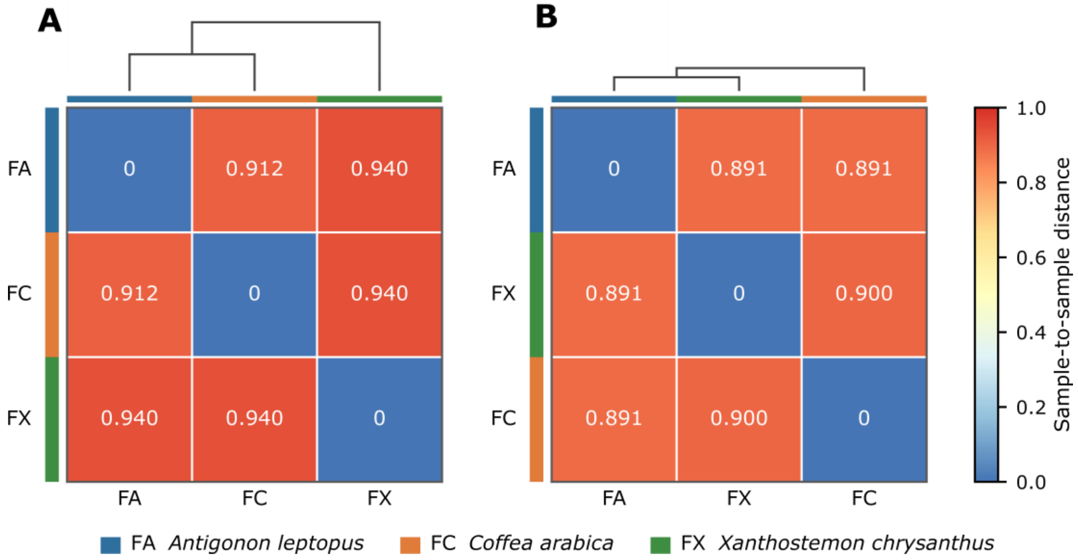

Figure S7. Sample-distance clustering heatmaps. (**A**) Bacterial and (**B**) fungal communities. Color indicates pairwise distance between host flowering species, from low (blue) to high (red).

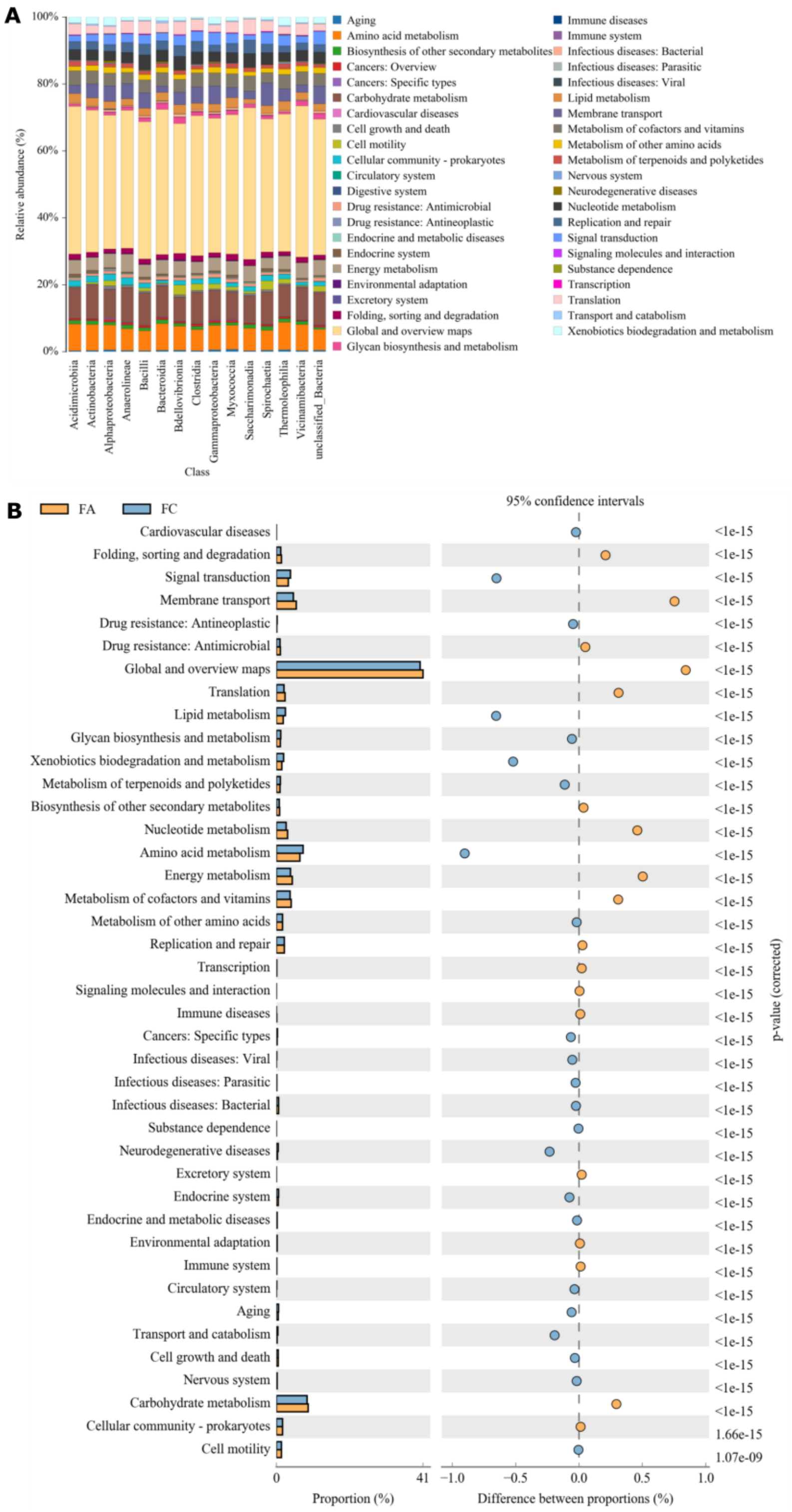

Figure S8. Predicted KEGG functional profiles of floral bacterial communities. (**A**) Relative abundance of predicted KEGG pathway categories across bacterial classes. (**B**) Comparison of predicted categories between *A. leptopus* and *C. arabica*, showing proportional abundance, difference between proportions and 95% confidence intervals.

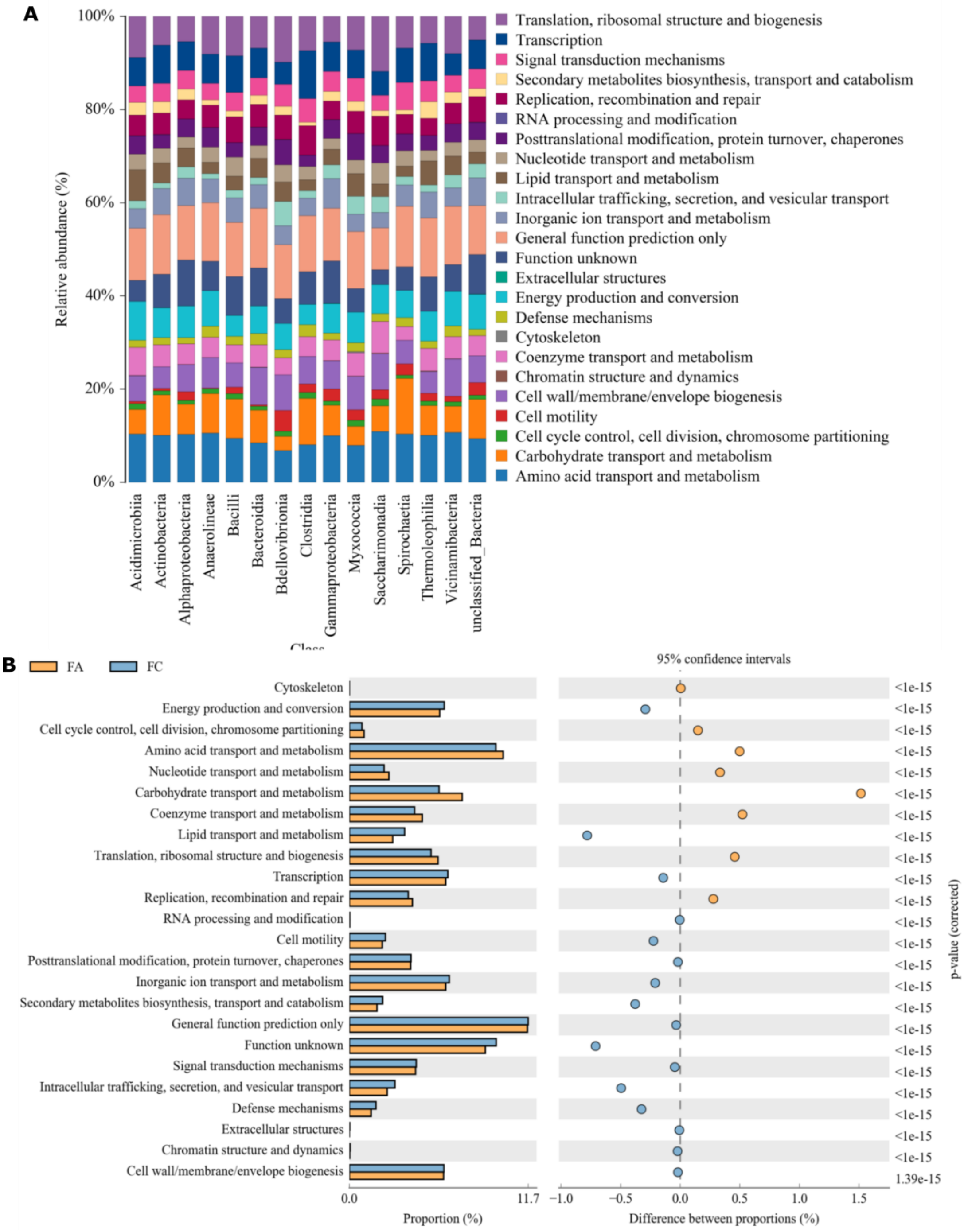

Figure S9. PICRUSt2-predicted COG functional profiles of floral bacterial communities. (**A**) Relative abundance of predicted COG functional categories across bacterial classes. (**B**) STAMP comparison of predicted COG categories between the *A. leptopus* and *C. arabica* libraries.

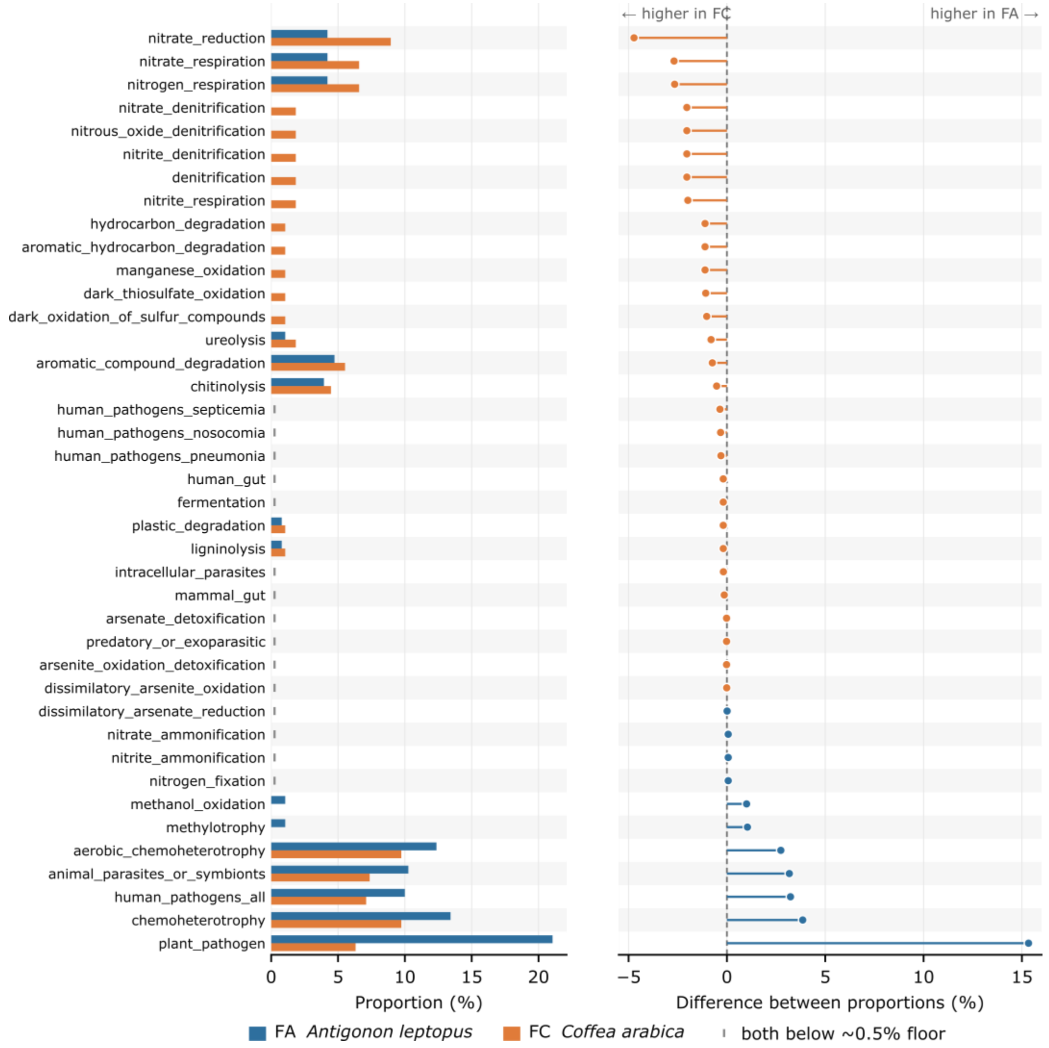

Figure S10. Predicted ecological functions of floral bacterial communities. FAPROTAX-inferred functional categories compared between *A. leptopus* and *C. arabica*, showing proportional abundance, difference between proportions and 95% confidence intervals.

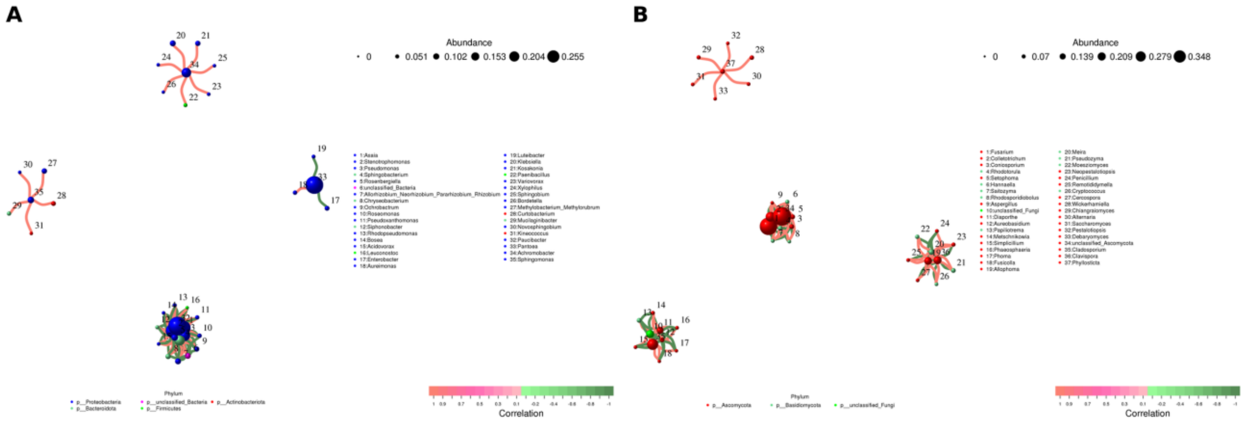

Figure S11. Spearman correlation networks among dominant genera. (**A**) Bacterial and (**B**) fungal communities. Node size scales with relative abundance, node color indicates phylum and edge color indicates the sign of the correlation. With three composite samples per marker gene, this display summarizes co-abundance among genera and does not constitute evidence of microbial interaction.
